# Assessment of nursing undergraduate's perceptions of Interprofessional learning: A cross-sectional study

**DOI:** 10.3389/fpubh.2022.1030863

**Published:** 2023-01-09

**Authors:** Adel S. Bashatah

**Affiliations:** Department of Nursing Administration and Education, College of Nursing, King Saud University, Riyadh, Saudi Arabia

**Keywords:** perceptions, readiness, Saudi students, nursing, patient care

## Abstract

**Background:**

Inter-Professional Learning (IPE) is based on mutual respect, and it improves collaboration and teamwork, and satisfaction among students and professionals.

**Objective:**

This study aimed to assess the perceptions of IPE among Nursing students in Saudi Arabia.

**Methods:**

This is a cross-sectional survey-based study conducted among students from three different universities in Saudi Arabia, among nursing students over 6 months from May 2021 to October 2021. Descriptive analysis was used to assess the perceptions of IPE and inferential testing was used to assess the association of perception scores among variables using a statistical package for social science version 26 (SPSS).

**Results:**

A total of 517 participants responded to the questionnaires. A higher proportion (*n* = 281, 54.4%) of the participants were females and were between 21 and 24 (*n* = 350; 67.7%) years old. The mean age of the participants was 21.35 (SD = 1.46). The majority of them were from King Saud University (*n* = 273, 52.8%), followed by King Khalid University (*n* = 127, 24.6%). Of the participants (80.4 %) agreed learning with other students will help them to become more effective members of a healthcare team. The mean overall score for RIPLS was 70.85 (SD = 6.611). The mean score for teamwork and collaboration was 37.19 (SD = 4.79), professional identity, 23.23 (SD = 2.89), roles and responsibilities 10.42 (SD = 2.20). The mean score is significantly associated with the university type (*p* = 0.0001), and previous knowledge of IPE (*p* = 0.0001).

**Conclusion:**

The majority of the students had positive perceptions of understanding IPE and a good level of preparation for IPE. This means that if IPE is conducted among Saudi students, students will benefit from it, and it has the potential to improve their capacity to deliver holistic nursing care to their patients.

## Introduction

In recent days Interprofessional education (IPE) is an important part of a healthcare student's education. Interprofessional learning involves students from two or more professions, who may learn together during their professional education, to establish a collaborative practice for providing patient-centered care (1–7) by analyzing their needs and interests through IPE for professional success, learning gaps between health education and practice settings are eliminated (8). Although IPE differentiates multi-professional education from shared and common learning (9). In contrast to traditional education, IPE focuses on providing the knowledge and trainee skills needed for collaborative teamwork (4, 10). Notably, IPE should be incorporated into the curriculum of healthcare students for achieving better health outcomes through collaborative teamwork.

According to World Health Organization IPE is defined as “when students from two or more professions learn about, from, and alongside each other to enable successful collaboration to improve the health outcomes (11, 12). Earlier studies from other countries revealed IPE allows students to learn about various professions, create positive attitudes, and acquire cooperative teamwork through social engagement with other disciplines (13–15).

Interprofessional collaborative practice has also been shown to be a key to better patient-centered, efficient, and cost-effective care, as well as a reduction in error rates (16, 17). Despite the team-oriented nature of the IPE, literature additionally suggested that IPE improves staff communication and interactions, as well as patient outcomes (17–20). Interprofessional education has several advantages in terms of academics and health outcomes (12).

Implementation of IPE has been shown to boost job satisfaction and minimize conflict and tension in the workplace (10, 21). The utilization of IPE has aided students in developing high-level knowledge, abilities, and professional attitudes to deal with the complexities of clinical circumstances in a collaborative and Interprofessional manner (22) Countries such as the United States, the United Kingdom, Canada, and European countries have effectively implemented IPE for nearly two decades, whereas Interprofessional education is only now being created in many other countries, including Saudi Arabia (23–25).

According to earlier studies, many international universities showing interest in IPE (26–29). An earlier study by Pollard et al., among British university students, assessed students' perspectives on IPE and found that they have positive attitudes toward IPE (28). Similarly, another study by Lumague et al. stated that healthcare students who have had Interprofessional experience understand the value of Interprofessional teamwork in inpatient care (29). Similarly, another recent study done on professors of medical, nursing, and pharmacy schools in South Korea reported that 85.2% of studied subjects were unaware of IPE (30). In 2016 Zeeni et al. assessed students' perceptions of IPE and found that students' readiness for IPL improved after participating in the IPE program. Additionally, students were satisfied with their learning experience, and assessment results revealed that all of the IPE learning objectives had been met (31). In Saudi Arabia there is a dearth of literature on the perceptions of students toward IPE, therefore we aimed this study to evaluate the perceptions of nursing students toward IPL in Saudi Arabia.

## Methods

### Study design, setting, and population

A cross-sectional web-based study was conducted in three different Saudi universities namely king Saud, King Khalid, and Taif universities in Saudi Arabia from May to October 2021 using structured validated self-administered questionnaires. All the undergraduates who were enrolled in the nursing curriculum in Saudi universities were included. Before data collection ethical approval was obtained from the college of medicine, King Saud University Riyadh, Saudi Arabia (Reference No. UQU-COP-EA #143706). Before carrying out the study verbal informed consent was obtained from the participants, and the participants were assured that the data would be used only for research and confidentiality would be maintained throughout the study. Moreover, this research study followed the principles of the Declaration of Helsinki 1995 (32).

### Sample size

There were ~2,000 residential students currently enrolled in nursing courses at Saudi universities in Saudi Arabia. Similar to the previous studies (33–38) we calculated the required sample size using the Raosoft sample size calculator (http://www.raosoft.com/samplesize.html.) with a 95% CI and a pre-determined margin of error of 5%. Because we were unaware of the potential results for each question, we assumed that the response distribution for each question would equal 50%. Although the sample size was projected to be 132, we opted to poll at least 200 students to assure greater reliability.

### Questionnaire design

An Arabic version of a RIPLS questionnaire for this study was used based on a previously published study by Bashatah et al. (39). The original version of the Readiness for IPL Scale (RIPLS) was published by Parsell and Bligh (40). The original questionnaire was in English language and was translated into Arabic language. The translation of the questionnaires was done by using forwards and backward procedures (41). The prepared questionnaire was subjected to face and content validity by two academics with extensive experience in preparing research questionnaires.

The questionnaire was modified in light of the feedback received from the experts. The final version of the questionnaires consisted of 25-items divided into four sections. The first section collected data on the demographic characteristics of the participants, including age, type of university, and level of education, the second section collected information on the perception of students about the Readiness for Inter-Professional Learning Scale (RIPLS) with a total of 19-items divided into three domains namely teamwork and collaboration (items 1–9), professional identity (items 10–16), and roles and responsibility (items 17–19). All these questionnaires were accessed student's perceptions on a five-point Likert scale. (1 = strongly disagree, 5 = strongly agree). The mean scores were calculated for each of the items in the scale, the total mean scores were further computed by combining all the item scores.

The data was gathered from the target population's using a convenience sampling approach. An online survey was used to gather the data. The electronic link was built using the Google forms we created. To determine the point of contact for the targeted population for data collection, we first spoke with the course instructor. The online poll used social media (WhatsApp). The study title was followed by a revealing statement, consent, and authorization to utilize completed information for publishing at the beginning of the survey. The students were informed that their participation was voluntary and anonymous, and those who read the following page and nodded in agreement were given the go-ahead to answer the research questions on it.

### Data analysis

The collected data were analyzed using the IBM SPSS Statistics 26 (IBM Inc., Chicago, IL, USA) and IBM SPSS 22 (IBM Inc., Chicago, IL, USA) software. Descriptive statistics were used for the demographic variables. Descriptive statistics such as percentages, frequency, and mean values were calculated. The mean scores of the RIPLS were compared between the demographics and other characteristics of the participants. A one-sample *t*-test and one-way ANOVA were used to compare the mean scores, and the results were considered statistically significant if the *p*-value was < 0.05.

## Results

A total of 517 participants responded to the questionnaires. A higher proportion (*n* = 281, 54.4%) of the participants were females and they were between the age of 21–24(*n* = 350; 67.7%), (mean age 21.35 ± 1.46) while the majority of them were from king Saud university (*n* = 273, 52.8%), followed by king Khalid university (*n* = 127, 24.6%) and Taif University (*n* = 117, 22.6%). Concerning the academic level, most of the students are in their second level (*n* = 124, 24%), sixth level (*n* = 99, 19.1%), fourth level (*n* = 83, 16.1%), Fifth level (*n* = 60, 11.6%), and internship (*n* = 30, 5.8%). Regarding previous knowledge of IPL 65.4 % of the students did not know about this. The summary of student information is presented in Table 1.

**Table 1:** Demographic characteristics of participants.

**Variable**	**Frequency (*n*)**	**Percentile (%)**	**Mean ±SD**
**Gender**			
Male	236	45.6	
Female	281	54.4	
**Age (years)**			
18–20	159	30.8	
21–24	350	67.7	21.35 ± 1.46
>25	8	1.5	
**Name of university**			
King Saud University	273	52.8	
Taif University	117	22.6	
King Khaled University	127	24.6	
**Academic level**			
First level	46	8.9	
Second level	124	24	
Third level	37	7.2	
Fourth level	83	16.1	
Fifth level	60	11.6	
Sixth level	99	19.1	
Seventh level	31	6	
Eighth level	6	1.2	
Internship	30	5.8	
**Do you have previous knowledge of Interprofessional learning?**			
Yes	178	34.4	
No	338	65.4	
**Source of knowledge**			
No	279	54	
From KSU	67	13	
Out of KSU	9	1.7	
From TU	21	4.1	
Out of TU	7	1.4	
From KKU	66	12.8	
Out of KKU	8	1.5	

Table 2 illustrates the perception of knowledge of IPE among students. Out of 19 items, students disagreed with items no 10, 11, 12, and 18 which illustrated the gaining of knowledge from others. Of the participants, 80.4% agreed that learning with other students would help them to become more effective members of a healthcare team. In addition, 80.1% agreed with item 2 which is to solve patient problems, healthcare students should work together. While 64.3% agreed, shared learning with other healthcare students would increase their ability to understand clinical problems. When the students were asked about learning with healthcare students would improve their relationships after qualification (76.2%) agreed to this item.

**Table 2:** Frequency of responses toward perception of IPE among university students.

**Items**	**Strongly disagree *n* %**	**Disagree *n* %**	**Neutral *n* %**	**Agree *n* %**	**Strongly agree *n* %**	**Mean ±SD**
Q1. Learning with other students will help me become a more effective member of a healthcare team	2 (0.4)	9 (1.7)	90 (17.4)	163 (31.5)	253 (48.9)	4.27 ± 0.835
Q2. Patients would ultimately benefit if healthcare students worked together to solve patient problems	11 (2.1)	2 (2.0)	92 (17.8)	209 (40.4)	205 (39.7)	4.18 ± 0.794
Q3. Shared learning with other healthcare students will increase my ability to understand clinical problems	1 (0.2)	10 (1.9)	174 (33.7)	190 (36.8)	142 (27.5)	3.89 ± 0.834
Q4. Learning with health-care students before qualification would improve relationships after qualification	1 (0.2)	13 (2.5)	109 (21.1)	163 (31.5)	231 (44.7)	4.18 ± 0.863
Q5. Communication skills should be learned with other healthcare students	1 (0.2)	19 (3.7)	73 (14.1)	217 (42)	207 (40)	4.18 ± 0.822
Q6. Shared learning will help me to think positively about other professionals	1 (0.2)	17 (3.3)	106 (20.5)	223 (43.1)	170 (32.9)	4.05 ± 0.825
Q7. For small-group learning to work, students need to trust and respect each other	1 (0.2)	2 (0.4)	65 (12.6)	167 (32.3)	280 (54.2)	4.41 ± 0.741
Q8. Team-working skills are essential for all healthcare students to learn	4 (0.8)	19 (3.7)	97 (18.8)	205 (39.7)	192 (37.1)	4.09 ± 0.877
Q9. Shared learning will help me to understand my limitations	3 (0.6)	28 (5.4)	131 (25.3)	188 (36.4)	167 (32.3)	3.94 ± 0.919
Q10. I don't want to waste my time learning with other healthcare students	113 (21.9)	214 (41.4)	139 (26.9)	37 (7.2)	14 (2.7)	2.27 ± 0.971
Q11. Undergraduate healthcare students don't need to learn together	115 (22.2)	204 (39.5)	145 (28)	39 (7.5)	14 (2.7)	2.29 ± 0.983
Q12. Clinical problem-solving skills can only be learned with students from my department	121 (23.4)	169 (32.7)	148 (28.6)	46 (8.9)	33 (6.4)	2.42 ± 1.129
Q13. Shared learning with other healthcare students will help me to communicate better with patients and other professionals	0 (0)	21 (4.1)	106 (20.5)	187 (36.2)	203 (39.3)	4.11 ± 0.866
Q14. I would welcome the opportunity to work on small-group projects with other healthcare students	3 (0.6)	19 (3.7)	117 (22.6)	225 (43.5)	153 (29.6)	3.98 ± 0.850
Q15. Shared learning will help to clarify the nature of patient problems	0 (0)	10 (1.9)	106 (20.5)	250 (48.4)	151 (29.2)	4.05 ± 0.757
Q16. Shared learning before qualification will help me become a better team worker	0 (0)	17 (3.3)	109 (21.1)	188 (36.4)	203 (39.3)	4.12 ± 0.850
Q17. The function of nurses and therapists is mainly to provide support to doctors	41 (7.9)	43 (8.3)	113 (21.9)	209 (40.4)	111 (21.5)	3.59 ± 1.147
Q18. I'm not sure what my professional role will be	93 (18.0)	141 (27.3)	140 (27.1)	98 (19.0)	45 (8.7)	2.73 ± 1.209
Q19. I have to acquire much more knowledge and skills than other healthcare students	2 (0.4)	23 (4.4)	117 (22.6)	154 (29.8)	221 (42.7)	4.10 ± 0.926

In this study majority of the students (82%) agreed that Communication skills should be learned with other healthcare students. a large number of participants (76%) suggested that Shared learning will help them to think positively about other professionals. A majority of the students (86.5%) believed that for small group learning to work, students need to trust and respect each other. The majority of 76.8% agreed that Team-working skills are essential for all healthcare students to learn. About two-thirds (68.7%) of the students accepted item 9 which elucidated Shared learning helps them to understand their limitations. while (75.5%) of the participants agreed that Shared learning with other healthcare students would help them to communicate better with patients and other professionals. Most of the students in the study (73.1%) agreed that they would welcome the opportunity to work on small-group projects with other healthcare students.

Approximately (77.6%) of the students agreed that Shared learning will help them to clarify the nature of patient problems with a mean score of 4.05 (SD = 0.757). While (75.7%) agreed with Item 16 which stated that shared learning before qualification will help them become a better team worker with a mean score of 4.12 (SD = 0.850). About two-thirds (61.9 %) agreed that the function of nurses and therapists is mainly to provide support for doctors. Nearly three-quarters (72.5%) of students agreed that they have to acquire much more knowledge and skills than other healthcare students (45.3 %) of the students disagreed with the statement “I'm not sure what my professional role will be” (item 18). Detailed descriptions of the participant's responses to the perception of IPE were given in Table 2.

The overall mean score for RIPLS was 70.85 (SD = 6.611). The mean score for teamwork and collaboration was 37.19 (SD = 4.79), for professional identity, 23.23 (SD = 2.89), roles and responsibilities 10.42 (SD = 2.20) (Table 3). The mean score is significantly associated with the university type (*p* = 0.0001) and previous knowledge of IPE (*p* = 0.0001). However, there was no significant association between the mean score of IPE concerning gender and age group of the participants (*p* = 0.05). Furthermore, the association between the mean RIPLS score and with respected some characteristics of the students was given in Table 4.

**Table 3:** The mean score of RIPLS domains.

**Domains**	**Mean**	**Std**	**Range**
Teamwork and collaboration	37.191	4.791	24.0
Professional identity	23.236	2.898	19.0
Roles and responsibility	10.423	2.206	11.0
Total RIPLS score	70.851	6.611	48.0

**Table 4:** Gender, university type, and knowledge of IPE association with RIPLS score (*n* = 517).

**Variables**	**RIPLS-score**							
	**N**	**Mean**	**SD**	**SE**	**F**	***t***	**Mean square**	***P*-value**
**Gender**							
Male	236	70.47	7.29	0.474	7.188	−1.174	–	0.241^*^
Female	281	71.16	5.97	0.356				
**Do you have previous knowledge of Interprofessional learning?**							
Yes								
No	178	73.19	6.19	0.464	1.003	6.121	–	< 0.001^*^
	338	69.57	6.46	0.351				
**Age**							
18–20	159	71.06	7.31	0.57				
21–24	350	70.63	6.24	0.33	2.719	–	118.05	0.067^**^
>25	8	76	6	2.12				
**Name of university**							
King Saud University	273	71.71	6.92	0.418				
Taif University	117	68.02	6.3	0.583	14.549		604.18	< 0.001
King Khaled University	127	71.59	5.41	0.48				

^*^Independent sample *t*-test.

^**^ANOVA.

## Discussion

The educational system in Saudi Arabia has undergone an incredible transformation. Even though this is evidence that IPE is only used in academic courses in highly developed countries worldwide. In this new era of rapid development if the foundation of IPE and practice is established during students' campus years in classroom and simulation labs the healthcare system will gain high visibility of well-trained and well-qualified healthcare professionals (HCPs) that may have a great impact on health outcomes. This study is to determine the perception of IPE among students in Saudi Arabia. Our study results include the students of all academic levels from King Saud University, Taif University, and King Khaled University reported that only 34.4% of the students have previous knowledge of IPE whereas a study conducted among medical students of King Saud University Riyadh concluded that only 23.4% had a previous experience with IPE (11). Another study among health sciences faculties at the University of Sumatera Utara reported that 68% of the students stated that they had heard Interprofessional education (IPE) information (42). So, it makes a significant contribution that IPE should be made compulsory for all healthcare professionals.

It is noteworthy to mention that the Source of knowledge of IPE of the King Saud University students is only 13 %, while King Khalid university students scored 12.8 %. Our results concluded that a large proportion of the students believed that Learning with other students will help them to become more effective members of a healthcare team. This finding is consistent with a previous study published by King Abdul Aziz University (43). In this study, most of the students perceived that the Patients would ultimately benefit if healthcare students worked together to solve patient problems which are similar to a previous study published by Zechariah et al., in the United States (44), Hammick (4), Hammick (10). Overall, it is clear that healthcare professionals are the ones with whom patients interact most and will improve patient outcomes. So, there is a need for implementing IPE. Findings also reported that 64.3% of the students believed that Shared learning with other healthcare students will increase their ability to understand clinical problems. The results are consistent with studies conducted by Al-Qahtani in Dammam (45). In this study, we observed that most of the students support IPE. Learning with healthcare students before qualification would improve relationships after qualification IPE helps in for a successful professional career. The future health care is moving toward a more team-based approach so students will gain valuable clinical Interprofessional experience—working with students and providers from several disciplines to provide quality patient care and improve patient outcomes and experience and reduce workloads that cause burnout among healthcare professionals.

Our findings demonstrated that professional students, particularly those studying healthcare, benefit from collaborative learning since it improves their communication skills as well as their attitude toward other professionals. Similar results were reported by Ho JM et al., among nursing and physiotherapy students (46). Poor communication leads to errors and a negative patient experience. On the other hand, the psychological impact has a bigger impact on team-based learning collaboration (46, 47). However, the majority of students in the current survey (86.5%) felt that for small-group instruction to be successful, students needed to have mutual trust and respect. Team-based working will help students to address the emerging issues in health care, solve problems, and deliver services to population health which fosters a greater understanding of each profession's role in the care and health of patients as well as adds value and importance to each other and the patients. According to the current study, 72.5% of the students felt they needed to learn a lot more than other healthcare students did in terms of knowledge and abilities. According to other studies, medical students require a greater level of knowledge and expertise than nursing or pharmacy students (48). Overall, our data show that students' attitudes regarding IPE were favorable. Additionally, the goal of Interprofessional education was to offer a set of concepts and methods that could be evaluated, put into practice, and improved together.

### Future implications and limitations

To prevent poor outcomes during graduation and to obtain more reliable health outcomes through sharing of their knowledge and practice toward healthcare, undergraduate nurses must be aware of IPE. Understanding IPE and its advantages in the provision of healthcare will help to advance rational healthcare in the future. Future research is necessary to bridge knowledge gaps and debunk misconceptions regarding Interprofessional learning among aspiring professionals. There are certain limitations to the current study. First, the findings were based on a self-administered online questionnaire, which could have increased the risk of biases such as social desirability bias or recall bias. Second, the findings were based on a specific profession focusing only on nursing in Saudi Arabia, making them non-representative of other professions at both national and international levels and therefore not internationally applicable. Despite these limitations, our research proposes that more emphasis be placed on raising individual awareness of the IPL provided by professionals to improve the health outcomes in the community.

## Conclusion

In this study Students' perspectives on this IPE, experience is examined along with corresponding benefits and challenges. All participants in the study recognized the importance of Interprofessional teamwork in inpatient care and agreed that all healthcare education should include opportunities enabling them to develop the skills, behaviors, and attitudes needed for Interprofessional collaboration.

## Data availability statement

The original contributions presented in the study are included in the article/supplementary material, further inquiries can be directed to the corresponding author.

## Ethics statement

The studies involving human participants were reviewed and approved by college of medicine, King Saud University Riyadh, Saudi Arabia (Reference No. UQU-COP-EA #143706). The patients/participants provided their written informed consent to participate in this study.

## Author contributions

AB designed the study, prepared the proposal, supervised data collection, analyzed and interpreted the data, and drafted and prepared the manuscript.
